# Volumetric Modulated Arc Therapy Improves Outcomes in Definitive Radiochemotherapy for Anal Cancer Whilst Reducing Acute Toxicities and Increasing Treatment Compliance

**DOI:** 10.3390/cancers13112533

**Published:** 2021-05-21

**Authors:** Jacqueline Possiel, Hanne Elisabeth Ammon, Manuel Guhlich, Lena-Christin Conradi, Michael Ghadimi, Hendrik Andreas Wolff, Markus Anton Schirmer, Stephan Samel, Michael Mügge, Stefan Rieken, Martin Leu, Leif Hendrik Dröge

**Affiliations:** 1 Department of Radiotherapy and Radiation Oncology, University Medical Center Göttingen, Robert-Koch-Str. 40, 37075 Göttingen, Germany; jacqueline.possiel@med.uni-goettingen.de (J.P.); hanne.ammon@med.uni-goettingen.de (H.E.A.); manuel.guhlich@med.uni-goettingen.de (M.G.); mschirmer@med.uni-goettingen.de (M.A.S.); stefan.rieken@med.uni-goettingen.de (S.R.); martin.leu@med.uni-goettingen.de (M.L.); 2 Department of General, Visceral and Pediatric Surgery, University Medical Center Göttingen, 37075 Göttingen, Germany; lena.conradi@med.uni-goettingen.de (L.-C.C.); mghadim@uni-goettingen.de (M.G.); 3 University Medical Center Göttingen, 37075 Göttingen, Germany; h.wolff@strahlentherapie-muenchen.eu; 4 Department of Radiology, Nuclear Medicine and Radiotherapy, Radiology Munich, 80333 Munich, Germany; 5 Department of Radiotherapy and Radiation Oncology, University Medical Center Regensburg, 93053 Regensburg, Germany; 6 Praxis für Koloproktologie und chirurgische Endoskopie, Waldweg 1, 37073 Göttingen, Germany; stephan.samel@proktologie-goettingen.de (S.S.); muegge.michael@web.de (M.M.)

**Keywords:** anal cancer, radiochemotherapy, 3D conformal radiotherapy, volumetric modulated arc therapy, survival, acute toxicity, late toxicity, hematologic toxicity, treatment compliance

## Abstract

**Simple Summary:**

Intensity-modulated radiotherapy (IMRT) is the standard of care in definitive chemoradiotherapy (CRT) for anal cancer. Only a limited number of studies have analyzed the clinical results with VMAT (volumetric modulated arc therapy, the advanced form of IMRT). We conducted a retrospective study on patients treated at our institution. We compared the outcomes of VMAT-treated and 3DCRT (3D conformal radiotherapy)-treated patients. VMAT reduced acute toxicities (i.e., primarily dermatitis and enteritis) to a great extent. Additionally, VMAT relevantly improved treatment compliance (i.e., less CRT interruptions/delays, shorter overall treatment time, and higher absolute 5-fluorouracil dose applied). Finally, we found improved cancer-specific survival and distant control in VMAT-treated patients. The present study underlines the great progress that has been achieved with IMRT/VMAT in the CRT of anal cancer. Our study is the first to demonstrate an improvement in treatment compliance and outcomes with VMAT. Future studies could address whether VMAT is advantageous when compared to conventional IMRT.

**Abstract:**

Background: Intensity-modulated radiotherapy (IMRT) is the standard of care in chemoradiotherapy (CRT) for anal cancer. Until now, only a limited number of studies have analyzed the results with VMAT (volumetric modulated arc therapy). We conducted a retrospective study on patients treated at our institution. Patients and Methods: We included patients who received curative CRT for anal cancer. We compared VMAT-treated and 3DCRT (3D conformal radiotherapy)-treated patients. We analyzed toxicities (acute: CTCAE criteria; late: LENT/SOMA criteria), treatment compliance, overall survival, cancer-specific survival (CSS), distant control (DC), and locoregional control. Results: A total of 149 patients (3DCRT: *n* = 87, VMAT: *n* = 62) were included. The median follow-up was longer in 3DCRT-treated patients (3DCRT: 61.3 months; VMAT: 39.1 months; *p* < 0.05). VMAT-treated patients had more G3 tumors (3DCRT: 12/87 (13.8%); VMAT: 18/62 (29.0%), *p* < 0.001). VMAT reduced acute toxicities ≥grade 3 (3DCRT: *n* = 48/87 (55.2%); VMAT: *n* = 11/62 (17.7%), *p* < 0.001). VMAT improved treatment compliance (less interruptions/delays) (3DCRT: 37/87, 42.5%; VMAT: 4/62, 6.5%; *p* < 0.001), provided a shorter median overall treatment time (3DCRT: 41 days; VMAT: 38 days; *p* = 0.02), and gave a higher median absolute 5-fluorouracil dose (3DCRT: 13,700 mg; VMAT: 14,400 mg; *p* = 0.001). Finally, we found improved CSS (*p* = 0.02; 3DCRT: 81.9% at 3 years; VMAT: 94.1% at 3 years) and DC (*p* = 0.01; 3DCRT: 89.4% at 3 years; VMAT: 100.0% at 3 years) with VMAT. Summary: Our study is the first to demonstrate improved treatment compliance and outcomes with VMAT for anal cancer. Previous studies have indicated that organs at risk sparing might be more improved with the use of VMAT vs. with conventional IMRT. Future studies should address whether these advantages lead to a further reduction in CRT-associated morbidity.

## 1. Introduction

The incidence of anal cancer has constantly increased during the last few decades [1]. Deshmukh et al. reported a two-fold increase in the incidence of non-metastatic disease and a three-fold increase in the incidence of metastatic disease during the period 2001–2015 in the United States [2]. At the same time, mortality rates increased by 3.1% per year [2].

Definitive chemoradiotherapy (CRT) is the standard treatment for non-metastatic anal cancer [3]. In localized disease (T1–2, N0), survival rates of ≥80% can be achieved [3,4]. In locally advanced tumors (T3–4/N+), survival rates of about 60% require further improvements [3,4]. Recent studies have reported that locoregional recurrences are a major site of failure [5,6]. At the same time, definitive CRT is associated with high rates of acute and hematologic toxicities [7]. Long-term morbidity leads to a significant impairment of patients’ quality of life [8,9]. As a consequence of CRT-related toxicities, treatment interruptions or delays are necessary for a relevant proportion of patients [4,10]. These events can negatively affect oncologic outcomes [4,10].

In radiotherapy delivery techniques, numerous efforts have been made to optimize outcomes and reduce treatment-associated morbidity. These efforts include image-guided radiotherapy; proton therapy; and, the most important progress, intensity-modulated radiotherapy (IMRT, with volumetric modulated arc therapy (VMAT) as a special form) [11,12,13,14,15,16]. The favorable toxicity profiles of IMRT in anal cancer were demonstrated in the prospective RTOG 0529 trial [14]. Additionally, numerous retrospective studies have highlighted the beneficial effects of IMRT on treatment compliance and survival [17,18,19,20]. Treatment planning studies have indicated that VMAT might even improve organs at risk sparing in comparison to conventional IMRT [21,22,23]. However, a limited number of studies with only a few patients have provided clinical results with VMAT for anal cancer [24,25,26,27,28].

In our institution, radiotherapy for anal cancer has been delivered with VMAT since 2010. In a previous publication, we highlighted the technique’s potential to reduce acute toxicities in comparison to conventional 3D conformal radiotherapy (3DCRT) [27]. At that time, the limited follow-up prevented firm conclusions being drawn about late toxicities and patient survival [27]. Here, we present an updated and extended analysis of this cohort. We analyzed acute toxicities, treatment compliance, late toxicity, and patient survival with VMAT in comparison to 3DCRT.

## 2. Patients and Methods

### 2.1. Patient Eligibility, Staging Procedures, Treatment Strategies, and Ethical Approval

We included all patients who were treated in our clinic with definitive CRT for anal cancer with curative intent. A previous study already reported results for a subset of patients [27]. Patients with cases of distant metastatic spread were excluded. The pre-treatment staging examinations included a rectoscopy and/or colonoscopy, a chest radiograph and an abdominal ultrasound or a computed tomography of the chest and abdominal region. Additionally, pelvic MR imaging was acquired. PET/CT was not used for staging during the treatment period. In female patients with advanced tumors, a gynecological examination was performed. The treatment strategies were determined in the multidisciplinary tumor board of our cancer center. Pre-treatment assessment and therapeutic decisions were based on the national and international guidelines [29,30]. The study was conducted in accordance with the principles of the Declaration of Helsinki. It was approved by the ethical committee of the University Medical Center of Göttingen (protocol codes 17/1/21 and 41/3/21). Due to the retrospective study design, additional informed consent was not required.

### 2.2. Chemoradiotherapy

The CRT procedures have been described previously [27]. Patients received percutaneous radiotherapy to the primary tumor and to the mesorectal, iliac, and inguinal lymph nodes. A total dose of 50.4 Gy in 1.8 Gy fractions was applied as a standard. In individual cases (e.g., small primary tumors or very advanced tumors), the treating radiation oncologist prescribed different total doses. Treatment was performed according to national and international guidelines [29,30,31]. Radiotherapy was planned based on CT scans. Patients were standardly treated in a prone position. Additionally, they were instructed to present with a comfortably filled bladder. During the study period, the radiotherapy delivery techniques used were 3DCRT (with individualized treatment fields) and VMAT (RapidArc®, Varian Medical Systems, Palo Alto, USA, treatment planning system Eclipse). For the treatment planning of VMAT, the following organs at risk constraints were used: bladder ≥65 Gy/ ≤ 25% and ≥40 Gy/ ≤ 50%, small bowel ≥50 Gy/ ≤ 10 cm^3^ and ≥40 Gy/ ≤ 100 cm^3^; rectum ≥65 Gy/ ≤ 17% and ≥40 Gy/ ≤ 50% [32]. In 3DCRT planning, the dose exposure to organs at risk was evaluated individually and was left at the discretion of the treating physician.

The concomitant chemotherapy was administered on an outpatient basis or during inpatient stay. The pre-chemotherapy examinations included the assessment of a complete blood cell count, blood clinical chemistry, an electrocardiogram, and a lung function test. The concomitant chemotherapy standardly consisted of 5-fluorouracil (medac GmbH, 22,880 Wedel, Germany) (d1–4, d29–32, 1000 mg/m^2^ of body surface area/d) and mitomycin c (d1, d29, 10 mg/m^2^ of body surface area/d) [27,33]. In cases of medical contraindications (e.g., decreased pulmonary function), cisplatin was used instead of mitomycin c [34,35,36].

### 2.3. Toxicity Scoring and Patient Follow-Up

As described previously, patients were assessed at least weekly for acute toxicities [27]. Here, a complete blood cell count and the blood clinical chemistry were acquired. Toxicities were scored according to the CTCAE criteria (acute toxicities, current version 5.0 [37]) and according to the LENT/SOMA system (late toxicities, [38]). In the radiotherapy department, patient follow-up was conducted for 5 years. Additionally, patients regularly presented to their treating gastroenterologists, proctologists, or visceral surgeons for endoscopic evaluation. The follow-up examinations were performed in accordance with the contemporary guidelines [29,30].

### 2.4. Statistical Procedures

The statistical comparisons of treatment groups (3DCRT vs. VMAT) were performed using the chi-square test and Mann–Whitney U test. The survival probabilities were calculated with Kaplan–Meier statistics. They were compared between treatment groups with the log-rank test. The survival times were analyzed for OS (event: patient death, any cause), CSS (event: death caused by anal cancer), distant control (event: occurrence of distant metastasis), and LRC (events: local recurrence and pelvic/inguinal recurrence). SPSS (IBM, v. 26) and ‘R’ (v. 4.0.2 with ‘KMWin’ [39]) were used for the analysis and for generating the survival curves. *p*-values <0.05 were considered to be statistically significant.

## 3. Results

### 3.1. Baseline Patient Characteristics and Follow-Up

A total of 149 patients (*n* = 87 for 3DCRT and *n* = 62 for VMAT) were included in the present study. Patients were treated from 03/1992 to 05/2019. VMAT was used from 02/2010 onwards. During the period of VMAT implementation (02/2010–11/2012), 16 consecutive patients were treated with either 3DCRT (*n* = 10) or VMAT (*n* = 6). The last treatment with 3DCRT was started in 11/2012. Afterward, all consecutive patients were treated with VMAT. During the implementation period of VMAT, the decision of whether to use 3DCRT or VMAT was taken on an individual basis by the treating physician. The median patient age was 62.9 years (range, 29.5–90.9 years). The median follow-up was 61.3 months (range, 2.4–268.4 months) in patients treated with 3DCRT and 39.1 months (range, 2.8–106.2 months) in patients treated with VMAT (*p* < 0.05, Mann–Whitney U test). Additionally, the treatment groups significantly differed in terms of tumor grade, with more aggressive tumors found in VMAT-treated patients (G3, 29.0% vs. 13.8%). Please see Table 1 for further details.

### 3.2. Chemoradiotherapy Characteristics and Treatment Compliance

In all patients, >80% of the planned radiotherapy dose could be applied. A total of 139/149 patients (93.3%) received concomitant chemotherapy. The regimen was 5-fluorouracil/mitomycin c in 131/139 patients (94.2%). Additionally, four patients received 5-fluorouracil/cisplatin, one patient received cisplatin alone, one patient received 5-fluorouracil alone, and two patients received mitomycin c alone. The treatment compliance was significantly better in patients treated with VMAT (parameters: radiotherapy interruptions/delays, absolute dose of 5-fluorouracil applied, overall treatment time). Please see Table 2 for further details. Please see Table S1 for the radiotherapy doses prescribed for the primary tumor and the lymph nodes. Please see Table S2 for the distribution of radiotherapy doses depending on the tumor stage.

### 3.3. Acute Toxicities, Hematologic Toxicities, and Late Toxicities

Acute organ toxicities ≥grade 3 occurred in 59/149 patients (39.6%). Hematologic toxicities ≥grade 3 was noted in 32/149 patients (21.5%). Late gastrointestinal and urinary toxicities ≥grade 3 were documented in 13/149 patients (8.7%). Here, in 10 patients a permanent stoma was necessary due to the gastrointestinal late side effects. In two patients, high-grade cystitis was evident. One patient suffered from grade 3 proctitis without receiving a stoma during follow-up. VMAT was associated with significantly lower rates of overall acute organ toxicity, dermatitis, and enteritis. Furthermore, VMAT was associated with higher rates of thrombopenia. Please see Table 3 for further details.

### 3.4. Overall Treatment Outcome

**Figure 1:**
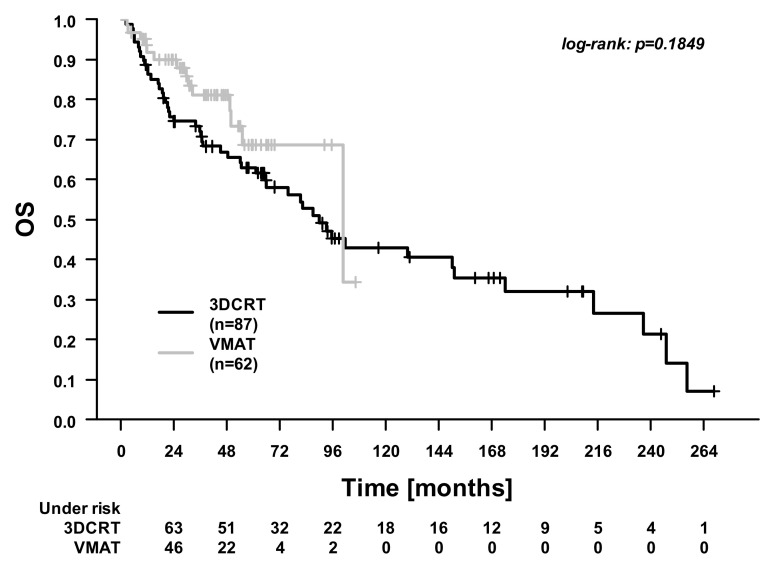
Overall survival (OS). Comparison between patients treated with 3D conformal radiotherapy (3DCRT) and patients treated with volumetric modulated arc therapy (VMAT).

**Figure 2:**
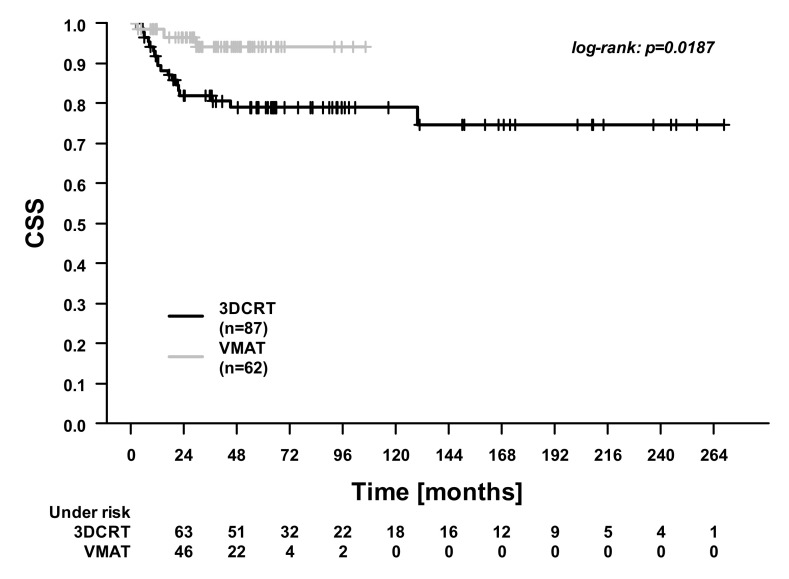
Cancer-specific survival (CSS). Comparison between patients treated with 3D conformal radiotherapy (3DCRT) and patients treated with volumetric modulated arc therapy (VMAT).

**Figure 3:**
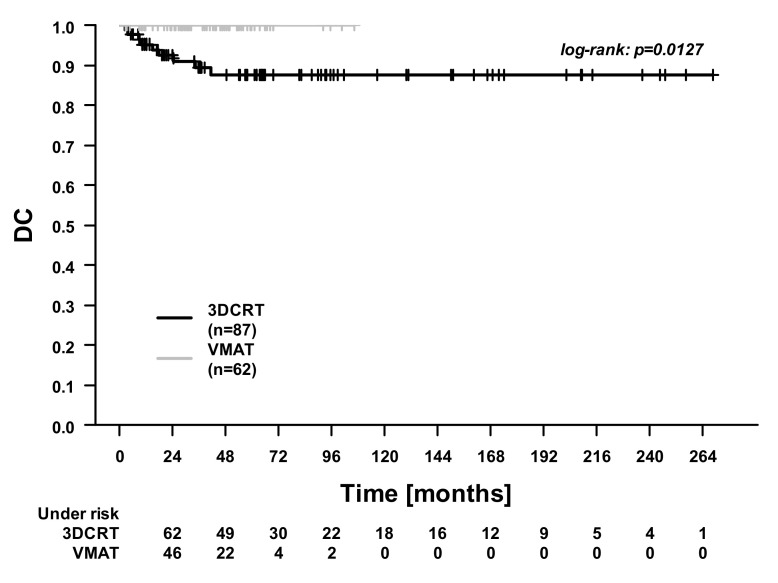
Distant control (DC). Comparison between patients treated with 3D conformal radiotherapy (3DCRT) and patients treated with volumetric modulated arc therapy (VMAT).

**Figure 4:**
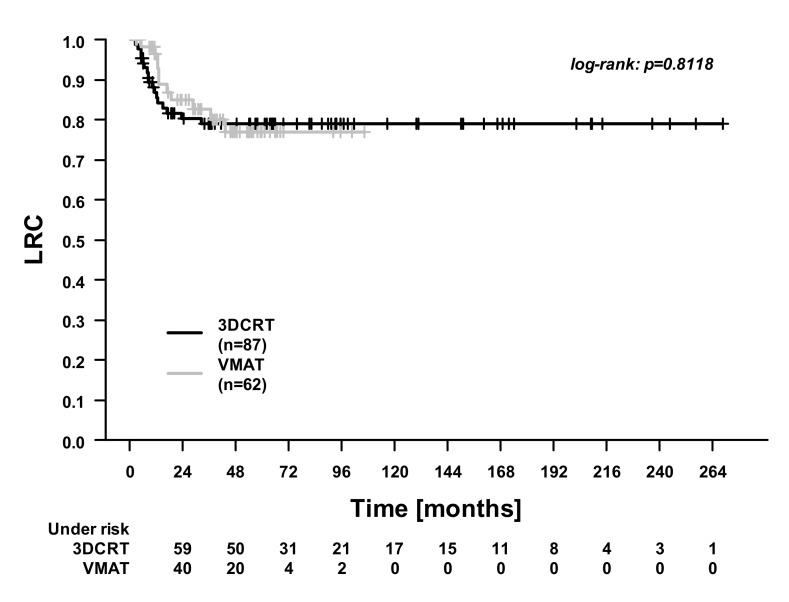
Locoregional control (LRC). Comparison between patients treated with 3D conformal radiotherapy (3DCRT) and patients treated with volumetric modulated arc therapy (VMAT).

In the whole cohort, the 5-year overall survival (OS), cancer-specific survival (CSS), distant control (DC), and locoregional control (LRC) were 74.9%, 86.8%, 93.6%, and 80.5%. Figure 1, Figure 2, Figure 3 and Figure 4 illustrate the OS, CSS, DC, and LRC for 3DCRT-treated and VMAT-treated patients. There were significant differences between the patients treated with 3DCRT and VMAT in CSS (*p* = 0.02) and in DC (*p* = 0.01). At 3 years, CSS was 81.9% for 3DCRT and 94.1% for VMAT, while DC was 89.4% for 3DCRT and 100.0% for VMAT. Among the nine patients with distant recurrences during follow-up, the sites of metastases were the lung (two patients), lung and pleura (one patient), lung and liver (two patients), liver (two patients), and bone (two patients).

## 4. Discussion

Definitive CRT is the standard treatment for anal cancer [3]. Especially in locally advanced tumors, the cure rates remain suboptimal and locoregional recurrences are a frequent site of failure [3,4]. Additionally, distant metastases occur in about 20% of patients during follow-up [6]. Radiotherapy delivery with IMRT leads to a reduction in CRT-related toxicities, to better treatment compliance, and to better oncologic outcomes [11,14,17,18,19,20,40]. Thus, IMRT is the standard of care in the radiotherapy of anal cancer [30]. VMAT (the rotational form of IMRT) was demonstrated to even further improve organ at risk sparing in dosimetric studies [21,22,23]. Until now, only a limited number of small studies have analyzed the clinical results with VMAT [24,25,26,27,28]. Martin et al. performed a patterns of care study in Germany, Austria, and Switzerland. Here, about 25% of the radiation oncology units used IMRT and about 70% used VMAT [41]. Thus, the clinical results with VMAT for anal cancer remain of significant interest for clinicians. We compared patients treated with VMAT and 3DCRT at our institution. Acute toxicities and survival rates with short-term follow-up were already presented in 2015 [27]. Herein, we present an updated and extended analysis including treatment compliance, late toxicity, and patient survival.

In our study, VMAT significantly reduced the rates of acute organ toxicities ≥grade 3. These events occurred in 48/87 patients (55.2%) who were treated with 3DCRT and only in 11/62 patients (17.7%) who were treated with VMAT. In accordance with previous studies, with rates of <20% for ≥grade 3 acute organ toxicities this underlines the potential of VMAT to relevantly reduce CRT-related morbidity [26,27,28]. In detail, we found a significant reduction in the rates of high-grade radiation dermatitis and high-grade enteritis. Both have already been reported in the comparison of conventional IMRT and 3DCRT [14]. Small studies indicated that bowel sparing might even be improved with VMAT in comparison to IMRT [22,23]. Thus, a comparison of the clinical results with IMRT and VMAT is of relevant interest. So far, clinical studies on this issue are lacking. For IMRT, previous studies reported ≥grade 3 skin toxicities in 21–38% of patients and ≥grade 3 gastrointestinal toxicities in 7–15% of patients [18,42,43]. In the present study, the rates for dermatitis and gastrointestinal toxicities were 15% and 3%, respectively (a combination of the parameters enteritis and proctitis), in patients treated with VMAT. Hence, the dosimetric advantages of VMAT over IMRT might translate into improved toxicity profiles [22,23]. However, a detailed comparison of the dose exposure to organs at risk between 3DCRT-treated and VMAT-treated patients was not included in the present analysis. This is an important limitation of our study. Nevertheless, the present study is the largest to highlight the clinical benefit of VMAT to reduce acute skin and bowel toxicity [27,28].

Next, we found an improvement in treatment compliance with VMAT. Before the IMRT era, treatment interruptions were necessary in up to 50% of all patients treated with CRT for anal cancer [44]. Accordingly, in the present study these events occurred in 42.5% of patients treated with 3DCRT. Previous studies have demonstrated a great advantage of IMRT over 3DCRT in terms of the rates of treatment interruptions/delays [14,18]. Additionally, they demonstrated a shortening of the overall treatment time [14,18]. In patients treated with IMRT, the rates of breaks were around 30% [17,18]. We present an even lower rate, with treatment interruptions/delays in only 6.5% of patients treated with VMAT. Furthermore, we found a reduction in overall treatment time in patients treated with VMAT. This emphasizes the great progress that has been achieved with the implementation of IMRT/VMAT in the CRT of anal cancer [26,45]. Additionally, in our study, VMAT-treated patients received a higher absolute dose of 5-fluorouracil than 3DCRT-treated patients. As Rich et al. pointed out, optimized radiotherapy techniques play a relevant role in further improving toxicity profiles in simultaneous CRT with 5-fluorouracil [46]. The low rates of toxicities with VMAT enable the achievement of higher chemotherapy intensity. Nevertheless, these findings should be interpreted with caution. The present study is of a retrospective design and included patients over a period of 27 years. Other changes in treatment practice, which were not evident in our study, might have influenced the outcome parameters. Progress in supportive management might have had a positive influence on toxicity rates and CRT compliance. However, our study is first to demonstrate an improvement in treatment compliance with VMAT in comparison to 3DCRT [24,25,26,27,28]. 

Moreover, VMAT-treated patients experienced better outcomes (CSS and DC) than 3DCRT-treated patients. Remarkably, we observed this effect in spite of the more aggressive tumors (G3 tumors, 29.0% vs. 13.8%) seen in the VMAT group [47]. During the last few decades, several studies have highlighted the negative prognostic impact of decreased treatment compliance on patient survival in anal cancer [4,10,44]. Thus, the better prognosis in VMAT-treated patients might be a consequence of the reduction in acute toxicities and the improvement of treatment compliance. A better survival rate in anal cancer patients has previously been reported when comparing IMRT to 3DCRT [18]. Bazan et al. found an improvement in local control, progression-free survival, and overall survival [18]. With very limited numbers of patients (here, a total of 64 patients), previous studies found no differences in survival when comparing VMAT and 3DCRT [28]. In the present study, we found a significantly better distant control in patients treated with VMAT. It can be hypothesized that the higher absolute dose of 5-fluorouracil might have improved the systemic control. However, survival outcomes should be cautiously interpreted. In the present study, we have a significant imbalance in follow-up between treatment groups. It is known that the vast majority of anal cancer recurrences occur within 3 years [30]. Hence, our study’s median follow-up (3DCRT: 61 months; VMAT: 39 months) can be considered sufficient. Overall, our study is the first to demonstrate an improvement in survival endpoints with VMAT compared to 3DCRT in anal cancer [24,25,26,27,28].

Furthermore, there was a tendency towards lower rates of ≥grade 3 late gastrointestinal and urinary toxicities with VMAT, reducing the percentage of affected patients by three quarters (*p* = 0.05; VMAT: 3.2%; 3DCRT: 12.6%). However, statistical significance was not reached in view of the relatively low event numbers. It can be expected that better organ at risk sparing and a reduction in acute toxicities could improve the late toxicity profiles [48,49]. However, the rates of late toxicities after irradiation with 3DCRT are already very low. This might render it difficult to discriminate further improvements. 

Finally, we found an increased rate of ≥grade 3 thrombopenia in patients treated with VMAT. Our study’s rate of high-grade thrombopenia (14.5% of the VMAT-treated patients) can be compared to that of previous studies. Goodman et al. reported that 16% of patients treated with CRT using IMRT and 5-fluorouracil/mitomycin c experienced ≥grade 3 thrombopenia [50]. Additionally, they demonstrated that infusional 5-fluorouracil, when compared to capecitabine, is associated with higher rates of hematologic toxicities [50]. In our study, VMAT-treated patients received a higher absolute dose of 5-fluorouracil than 3DCRT-treated patients. The higher chemotherapy intensity could explain the higher rates of hematologic toxicities. Moreover, it has been demonstrated that bone marrow sparing in IMRT/VMAT planning is able to reduce hematologic toxicities in anal cancer [15,51]. In our study, this was not implemented in the planning process. Thus, the integration of bone marrow sparing should be advocated to further reduce hematologic toxicities of CRT in patients with anal cancer.

## 5. Conclusions

IMRT is the standard of care in definitive CRT for anal cancer. Until now, only a limited number of small studies have analyzed the clinical results with VMAT (the advanced form of IMRT). We conducted a retrospective study on patients treated with CRT for anal cancer at our institution. We compared the outcomes in VMAT-treated and 3DCRT-treated patients. VMAT reduced acute toxicities (i.e., primarily dermatitis and enteritis) to a great extent. Additionally, VMAT relevantly improved the treatment compliance (i.e., less CRT interruptions/delays, shorter overall treatment time, and higher absolute 5-fluorouracil dose). Finally, we found improved outcomes (CSS and DC) in VMAT-treated patients. The present study underlines the great progress that has been achieved with IMRT/VMAT in the CRT of anal cancer. Our study is the first to demonstrate an improvement in the treatment compliance and outcomes with VMAT for anal cancer. Previous studies have indicated that organ at risk sparing might be improved with VMAT in comparison to conventional IMRT. Thus, future studies should address whether these advantages are reflected in a further reduction in CRT-associated morbidity.

## Figures and Tables

**Table 1:** Patient baseline characteristics.

Characteristics	3DCRT (*n* = 87)	VMAT (*n* = 62)	*p*-Value
Age (years)	64.3 (29.5–90.9)	60.2 (33.3–84.5)	0.11 #
Sex			0.92 *
Female	61 (70.1)	43 (69.1)	
Male	26 (29.9)	19 (30.6)	
Body Mass Index (kg/m^2^)	25.6 (14.1–44.6)	25.8 (17.4–38.5)	0.83 #
Body Surface Area (DuBois method, m^2^)	1.76 (1.31–2.22)	1.8 (1.45–2.46)	0.21 #
Charlson Comorbidity Index			0.54 *
1–3	27 (31.0)	23 (37.1)	
4–6	52 (59.8)	35 (56.5)	
7–10	8 (9.2)	4 (6.4)	
Grading			<0.001 *
G1	18 (20.7)	0	
G2	56 (64.4)	42 (67.7)	
G3	12 (13.8)	18 (29.0)	
Undetermined	1 (1.1)	2 (3.2)	
cT status			0.19 *
T1	14 (16.1)	12 (19.4)	
T2	40 (46.0)	20 (32.3)	
T3	26 (29.9)	19 (30.6)	
T4	7 (8.0)	11 (17.7)	
cN status			0.05 *
N0	60 (69.0)	33 (53.2)	
N1	27 (31.0)	29 (46.8)	
AJCC classification (8th edition, 2017)			0.06 *
I	11 (12.6)	10 (16.1)	
IIA	35 (40.2)	12 (19.4)	
IIB	11 (12.6)	6 (9.7)	
IIIA	7 (8.0)	10 (16.1)	
IIIB	3 (3.4)	6 (9.7)	
IIIC	20 (23.0)	18 (29.0)	

For each parameter, either the number and percentage or the median and range are given. For the comparison of treatment groups, we used the chi-square test * or the Mann–Whitney U test #. 3DCRT: 3D conformal radiotherapy. VMAT: volumetric modulated arc therapy.

**Table 2:** Chemoradiotherapy: characteristics and treatment compliance.

Parameters	3DCRT (*n* = 87)	VMAT (*n* = 62)	*p*-Value
Radiotherapy			
Planned dose	50.4 (40.0–61.0)	50.4 (50.4–60.4)	0.22 #
Administered dose	50.4 (40.0–61.0)	50.4 (41.4–59.4)	0.06 #
Received 100% of planned dose	73 (83.9)	57 (91.9)	0.15 *
Received >80% of planned dose	87 (100.0)	62 (100.0)	-
Interruptions or delays (patients)	37 (42.5)	4 (6.5)	<0.001 *
Interruptions or delays (days, mean, range) ^1^	2.65 (0.0–27.0)	0.26 (0.0–6.0)	<0.001 #
Chemotherapy			
Received concomitant chemotherapy	80 (92.0)	59 (95.2)	0.44 *
Chemotherapy regimen			0.06 *
5-fluorouracil/mitomycin c	78 (97.5)	53 (85.5)	
Other regimen	2 (2.5)	6 (14.5)	
Chemotherapy compliance and dose			
Interruptions or delays (patients)	5 (6.3)	1 (1.7)	0.19 *
Received <100% of planned dose	12 (15.0)	9 (15.3)	0.97 *
5-fluorouracil: absolute dose applied (mg) ^2^	13,700 (6000–15,000)	14,400 (6240–18,000)	0.001 #
Mitomycin c: absolute dose applied (mg) ^2^	34.6 (10.0–41.0)	35.6 (15.6–40.0)	0.78 #
Cisplatin: absolute dose applied (mg)	400 (1 patient)	255.75 (131.0–393.0)	-
Chemoradiotherapy			
Overall treatment time (days)	41 (28–74)	38 (31–49)	0.02 #

For each parameter, either the number and percentage or the median and range are given, if not otherwise specified. For the comparison of treatment groups, we used the chi-square test * or the Mann–Whitney U test #. 3DCRT: 3D conformal radiotherapy. VMAT: volumetric modulated arc therapy. ^1^ Interruptions or delays of radiotherapy: please note that information is missing for 1 patient. ^2^ Absolute doses of chemotherapy: please note that the information on absolute dose is missing for 7 patients (5-fluorouracil) and 8 patients (mitomycin c).

**Table 3:** Acute and hematologic toxicities (according to CTCAE criteria) and late toxicities (according to LENT/SOMA criteria).

Toxicities	3DCRT (*n* = 87)	VMAT (*n* = 62)	*p*-Value
Acute organ toxicity			
Overall acute organ toxicity, ≥grade 3	48 (55.2)	11 (17.7)	<0.001
Dermatitis, ≥grade 3	39 (44.8)	9 (14.5)	<0.001
Enteritis, ≥grade 3	9 (10.3)	1 (1.6)	0.04
Proctitis, ≥grade 3	4 (4.6)	1 (1.6)	0.32
Cystitis, ≥grade 3	3 (3.4)	2 (3.2)	0.94
Hematologic toxicity			
Overall hematologic toxicity, ≥grade 3	15 (17.2)	17 (27.4)	0.14
Anemia, ≥grade 3	2 (2.3)	1 (1.6)	0.77
Leukopenia, ≥grade 3	11 (12.6)	12 (19.4)	0.26
Thrombopenia, ≥grade 3	2 (2.3)	9 (14.5)	0.01
Late toxicity			
GI and urinary, ≥grade 3	11 (12.6)	2 (3.2)	0.05
Vagina, grades 1–2 ^1^	7 (13.0)	5 (12.2)	0.91
Pelvic bone fractures, ≥grade 3 ^2^	6 (6.9)	1 (1.6)	0.12

For each parameter, the number and percentage are given. For the comparison of treatment groups, we used the chi-square test. 3DCRT: 3D conformal radiotherapy. VMAT: volumetric modulated arc therapy. GI: gastrointestinal. ^1^ Vaginal toxicity includes vaginal dryness and dyspareunia. The information on vaginal toxicity was missing in 9/104 female patients. ^2^ The information on bone fractures was missing in 11 patients.

## Data Availability

The data that support the findings of this study are available from the corresponding author by reasonable request.

## Supplementary Materials

The following are available online at https://www.mdpi.com/article/10.3390/cancers13112533/s1: Table S1: Prescribed radiotherapy doses. Table S2: Prescribed radiotherapy doses and tumor stage.
